# Crown discoloration promoted by materials used in regenerative endodontic procedures and effect of dental bleaching: spectrophotometric analysis

**DOI:** 10.1590/1678-77572016-0398

**Published:** 2017

**Authors:** Luciane Geanini Pena dos SANTOS, Wilson Tadeu FELIPPE, Beatriz Dulcineia Mendes de SOUZA, Andrea Cristina KONRATH, Mabel Mariela Rodríguez CORDEIRO, Mara Cristina Santos FELIPPE

**Affiliations:** 1 Universidade Federal de Santa Catarina, Departamento de Odontologia, Florianópolis, Santa Catarina, Brasil.; 2 Universidade Federal de Santa Catarina, Departamento de Informática e Estatística, Florianópolis, Santa Catarina, Brasil.; 3 Universidade Federal de Santa Catarina, Departamento de Ciências Morfológicas, Florianópolis, Santa Catarina, Brasil.

**Keywords:** Regenerative endodontics, Spectrophotometry, Tooth discoloration, Triple antibiotic paste, Dental bleaching

## Abstract

Regenerative endodontic procedure (REP) has been proposed as a new approach to treat immature permanent teeth. However, materials used in REP for root canal disinfection or cervical sealing may induce tooth discoloration.

**Objectives:**

To assess tooth crown’s color after intracanal treatment with triple antibiotic paste (TAP) or calcium hydroxide (CH); cervical sealing with glass ionomer cement (GIC) or mineral trioxide aggregate (MTA); and bleaching with carbamide peroxide.

**Material and Methods:**

After pulp removal and color spectrophotometer measurement, 50 bovine incisors were divided into 4 experimental groups and one control (untreated). Experiments were performed in phases (Ph). Ph1: TAP (ciprofloxacin, metronidazole, minocycline), TAPM (ciprofloxacin, metronidazole, amoxicillin), DAP (ciprofloxacin, metronidazole), or CH treatment groups. After 1 and 3 days (d); 1, 2, 3 weeks (w); and 1, 2, 3 and 4 months (m), color was measured and medications were removed. Ph2: GIC or MTA cervical sealing, each using half of the specimens from each group. Color was assessed after 1d, 3d; 1w, 2w, 3w; 1m and 2m. Ph3: Two bleaching sessions, each followed by color measurement. Data were analyzed with ANOVA and post-hoc Holm-Sidak method.

**Results:**

Ph1: Specimens of TAP group presented higher color alteration (ΔE) mean than those of TAPM group. No significant difference was found among TAP or TAPM and CH, DAP or Control groups. Ph2: cervical sealing materials showed no influence on color alteration. Ph3: Different ΔE means (from different groups), prior to bleaching, became equivalent after one bleaching session.

**Conclusions:**

TAP induces higher color alteration than TAPM; color alteration increases over time; cervical sealing material has no influence on color alteration; and, dental bleaching was able to recover, at least partially, the tooth crown’s color.

## Introduction

Regenerative endodontic procedures (REP) have been proposed as an alternative to conventional endodontic treatment for immature permanent teeth, since it allows continuing root development in length and thickness^3,7,21,28^.

In *Clinical Considerations for a Regenerative Procedure*, American Association of Endodontists^2^ (AAE) advises root canal disinfection, blood clot formation and capping, cervical sealing, and crown restoration. For root canal disinfection, AAE^2^ recommends calcium hydroxide (CH) or triple antibiotic paste (TAP), consisting of ciprofloxacin, metronidazole and minocycline^12^. Although TAP provides good antibacterial effect, case reports have shown tooth staining induced by minocycline^9,16,23^. Thus, alternative medications have been proposed, as double antibiotic paste (DAP), composed by ciprofloxacin and metronidazole^24^, or TAP Modified pastes (TAPM) in which minocycline is replaced by other antibiotics, as amoxicillin^14,24^ or cefaclor^24,27^.

Subsequently to disinfection, AAE^2^advises a 3-4 mm layer of MTA, bioceramic, or glass ionomer cement (GIC) over the capped clot as cervical sealing. Since even white MTA could induce tooth discoloration^11,13^, alternatives to this material, such as bioceramics and GIC, have been strongly indicated in teeth where there is an esthetic concern^2^.

Although REP provides satisfactory biological results, products used in this approach could impair dental esthetic, which may not be reverted by tooth bleaching^16,20^. We did not find literature assessing tooth discoloration after each phase of REP, using different intracanal pastes and cervical sealing materials, followed by measuring color after bleaching. Therefore, investigation of alternatives to reduce tooth esthetic prejudice is justified. The aims of this study were to assess the effect of intracanal medications and sealing materials used in REP on tooth crown’s color, as well as on tooth bleaching response. The null hypotheses are: (1) materials used as intracanal dressing or cervical sealing, in REP, are not able to induce tooth crown discoloration; and, (2) in case of tooth discoloration promoted by those materials, dental bleaching is not able to recover tooth color.

## Material and methods

### Tooth selection and specimen preparation

Fifty bovine incisors with similar dentinal wall thickness were collected and disinfected by immersion in 1.5% sodium hypochlorite for 3 min. To simulate an immature tooth condition^6^ and standardize specimen length (15 mm), each tooth was sectioned 5 mm coronally and 10 mm radicularly from the cementum-enamel junction (CEJ) using a water-cooled diamond disc. After endodontic access, pulp tissue was removed and the root canal was enlarged with a 4103 diamond bur (KG Sorensen, Barueri, SP, Brazil) under irrigation with 3 mL of 1.5% NaOCl, to standardize the internal diameter in 1.6 mm and thickness of dentinal walls in 1.91*±*0.37 mm. The last radicular 5 mm were sealed with wax, and the root cervical third was irrigated with 3 mL of 1.5% NaOCl followed by 3 mL of 17% ethylenediaminetetraacetic acid (EDTA, Sigma-Aldrich, St. Louis, United States) to remove smear layer. Root canal was dried with absorbent paper points, a cotton pellet was placed inside the root canal and the crown was sealed with Citodur^®^ (DoriDent, Wien, Wien, Austria). During these procedures, teeth were wrapped up in gauzes embedded in distilled water to avoid dehydration; furthermore, after all preparations, they were stored submerged into 10 mL of distilled water.

### Spectrophotometry

**Figure 1:**
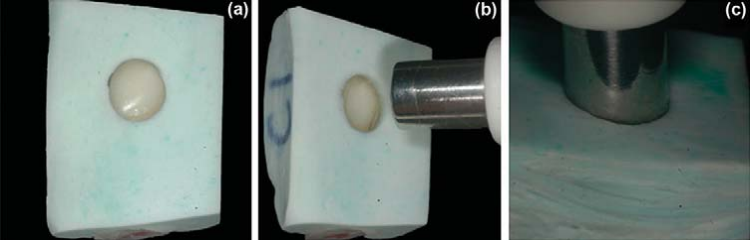
Tooth color assessment devices. (a) Custom silicone matrix covering the entire buccal tooth’s surface, (b,c) with a perforation to fit the spectrophotometer tip.

To standardize area and light conditions for color assessment, for each tooth, a custom silicone matrix was fabricated with impression material (Perfil^®^, Vigodent S/A, Rio de Janeiro, RJ, Brazil), covering the entire buccal tooth surface. A perforation compatible with the size of the spectrophotometer tip (*±*6 mm diameter) was made with a cutting-edge cylinder at the crown area^5^ (Figure 1a-c). Specimens’ colors were assessed using a digital spectrophotometer (Vita Easyshade^®^, VITA Zahnfabrik H. Rauter GmbH & Co.KG, Bad Säckingen, Baden-Württemberg, Germany), and following the *Commission Internationale de l’Éclairage* (CIE) Lab System, to obtain L* (lightness), a* and b* (hue) values.

The first color assessment was performed one day after specimens’ preparation and, before the assessment. For this, after removing specimens from distilled water, the excessive moisture of the external dental surface was removed with gauzes. After that, the experiment was performed in three phases:


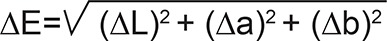


### Phase 1: Intracanal medication

Teeth were randomly divided into 1 control (root canal untreated) and 4 experimental groups (n=10), according to intracanal medication: TAP; TAPM (ciprofloxacin, metronidazole, and amoxicillin); DAP; and CH (Calen^®^, SS White, Rio de Janeiro, RJ, Brazil). Antibiotic pastes were prepared by the same pharmacy (Magistrale Farmácia de Manipulação, Florianópolis, SC, Brazil), at 0.1 mg/mL concentration and equal proportions of each antibiotic. All pastes were applied into the canals using syringe/needle (22 G1) (BD, Vernon Hills, IL, USA).

Coronal sealing was performed as previously described and teeth were stored once again in distilled water. After 1 and 3 days (d); 1, 2, and 3 weeks (w); and 1, 2, 3, and 4 months (m), specimens had the excessive moisture removed with gauzes and color shades were measured again.

Thereafter, intracanal medications were removed by saline solution irrigation and canals were dried with absorbent paper points for the next phase.

### Phase 2: Cervical sealing material

Specimens from each experimental group were randomly subdivided into 2 subgroups (n=5), according to the cervical sealing material: GIC (Vidrion R^®^, SS White) or White MTA (Angelus, Londrina, PR, Brazil). Following coronal sealing, the specimens returned to the distilled water. After 1d and 3d; 1w, 2w, and 3w; and 1m and 2m, color shades were registered again.

### Phase 3: Bleaching

Coronal sealing was removed to perform dental bleaching with 37% carbamide peroxide gel (BM4 - Brasil Materiais e Instrumentais, Palhoça, SC, Brazil). Teeth were inserted in a polyurethane sponge soaked with distilled water up to the CEJ. Bleaching gel was applied inside and outside dental crowns for 45 min, according to manufacturer’s recommendations. Gel was then removed by suction, specimens were washed in distilled water, crowns sealed and teeth wetly stored. Color shades were assessed after 24 hours. Two bleaching sessions were performed at 1-week interval.

### Obtainment of color alteration

For each specimen, the 3 parameters (ΔL, Δa, Δb) to measure color alteration (ΔE) were calculated subtracting the initial values from the final ones (ΔL=L2–L1, Δa=a2–a1, and Δb=b2–b1), at each time period. ΔE means for each group and experimental period were calculated using the following equation:

It is important to highlight that ΔE human perceptibility threshold was set at 3.7 units to determine which differences were clinically visible^1,29^. Therefore, any tooth color alteration higher than ΔE: 3.7 would be perceived by the human eye.

### Statistical analysis

Data were analyzed by Repeated Measures Analysis of Variance (ANOVA) and *post-hoc* Holm-Sidak method, at a significance level of 5%.

## Results

Supplemental Table 1 provides shades according to VITA Classical A1–D4^®^ Shade Guide from teeth throughout experiment.

**Supplemental Table 1:** Color shades of the samples according to VITA Classical A1–D4® Shade Guide throughout the experiment.

GROUP	SAMPLE	BASELINE	INTRACANAL MEDICATION				CERVICAL SEALING			BLEACHING		
			1w	1m	2m	4m	1w	1m	2m	SESSION1	SESSION2	
TAP	1	B1	B2	B2	B2	B2	B2	B2	B2	B2	A1	GIC
	2	A1	B2	B2	B2	B2	B2	B2	B2	B2	A1	
	3	A2	B2	B2	B2	B2	B3	B2	B2	B2	A1	
	4	B2	B2	B2	B2	B2	B3	B2	B2	A1	A1	
	5	A2	A2	A2	B2	B2	A2	B2	B3	A2	A2	
	6	A1	B2	B2	B2	B2	B3	B3	B3	A1	A1	MTA
	7	B1	B2	B2	B2	B2	B2	B3	B2	B2	B2	
	8	A2	A2	A2	A2	B2	B3	B3	B3	B2	B2	
	9	A1	B2	B2	B2	B2	B2	B2	B2	A1	B2	
	10	A2	A2	A2	A2	B2	B3	B3	B3	B2	B2	
TAPM	11	B2	B2	A1	A1	B2	B2	B2	B2	A1	A1	GIC
	12	A1	A1	A1	A2	A1	B2	B2	B2	A1	A1	
	13	B2	B2	B2	B2	B2	B2	B2	B2	B2	A1	
	14	A2	A2	A2	B2	B2	A2	A2	B2	B2	B2	
	15	A2	A2	A2	A2	A2	A2	A2	A2	B2	A1	
	16	A1	A1	A1	A1	A1	B2	B2	B2	B2	B2	MTA
	17	A3	A3	A3	A3	A3	B3	B3	B3	B3	B3	
	18	A2	A3	A2	A3	A2	A3	B3	B3	B3	B2	
	19	A2	A3	A3	A3	A2	B3	B3	B3	B3	B3	
	20	A1	A1	A1	B2	B2	B2	B2	B2	A1	A1	
DAB	21	A2	A2	B2	A2	A2	B3	B2	B3	A2	B2	GIC
	22	A1	A1	A1	B2	B2	B2	B2	B2	A1	B1	
	23	A1	A1	A1	B2	B2	B2	B2	B2	B1	B1	
	24	B2	B2	A2	A2	B2	B3	B2	B2	B2	A1	
	25	B2	B2	B1	B2	B2	B2	B2	B2	A1	A1	
	26	A3	A2	A3	B3	B3	B3	B3	B3	B3	B3	MTA
	27	A2	A2	A2	A2	A2	B2	B2	B3	B2	B2	
	28	A3.5	A3.5	A3.5	A.35	A3.5	A3.5	A3.5	B4	B3	B3	
	29	B2	B2	B2	B2	B2	B2	B2	B2	A1	A1	
	30	A1	A2	B2	A2	B2	B3	B2	B2	B2	B2	
CH	31	B1	B2	B2	B2	B2	B2	B2	B2	B2	A1	GIC
	32	A1	B2	B2	B2	B3	B2	B2	B2	A1	A1	
	33	A1	B2	B2	B2	B2	B2	B2	B2	B2	B2	
	34	B2	B2	B2	B2	B2	B3	B2	B2	B2	A1	
	35	A2	A2	B2	B2	B2	B2	B2	B2	B2	B2	
	36	B2	B2	B2	B2	B2	B2	B2	B2	B2	B2	MTA
	37	A1	B2	B2	B2	B2	B2	B2	B2	B2	B2	
	38	A2	B3	B3	B3	B3	B3	B3	B3	B2	B2	
	39	A2	A3	A3	B3	B2	B3	B3	B3	B3	B3	
	40	A2	A2	B2	B2	B2	B3	B2	B2	B2	B2	
Control	41	B2	B2	B2	B2	A1	B2	B2	B2	B2	B2	UT
	42	A1	A1	A1	B2	A2	B2	A1	A1	A1	A1	
	43	A2	A2	A2	A2	B2	B2	B2	B2	B2	B2	
	44	A1	A1	A1	C2	A1	B2	A1	B2	A1	A1	
	45	A2	A2	A1	A2	B2	B2	B2	B2	B2	B2	
	46	B2	B2	B2	A3	B2	B2	B2	B2	B2	B2	
	47	A1	A1	A1	B2	A1	B2	A1	A1	A1	A1	
	48	A2	A2	A2	B2	B2	B2	B2	B2	B2	B2	
	49	A1	A1	A1	A2	A2	B2	A1	B2	A1	B2	
	50	B2	A2	A2	B2	A1	B2	B2	B2	B2	B2	

CH: calcium hydroxide, DAP: double antibiotic paste, TAP: triple antibiotic paste, TAPM: TAP modified. GIC: glass ionomer cement, MTA: mineral trioxide aggregate, UT: untreated. d: day, m: month, w: week.

### Phase 1: Intracanal medication

**Figure 2:**
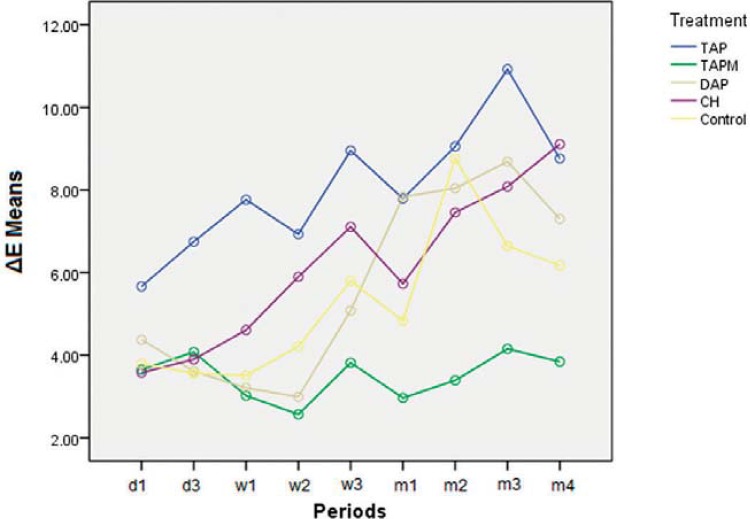
Graphic illustrating ΔE means from each group and period throughout Phase 1

As there was no significant difference in the interaction between treatment and periods (P=0.121), the results from these variables were presented separately (Table 1, Figure 2). Disregarding the period, TAP group presented the highest ΔE mean and there was a significant difference between TAP and TAPM groups. The latter showed ΔE mean below human perception threshold value. However, no significant difference was found among these pastes and CH, DAP, or Control.

**Table 1:** Mean values of ΔE and confidence intervals according to treatment and period of evaluation regarding Phase 1 (Intracanal medication)

Treatments	ΔE Mean
TAP M	3.5±0.5^a^
CONTROL	5.25±1.7^ab^
DAB	5.68±2.2^ab^
CH	6.16±1.9^ab^
TAP	8.06±1.5^b^
Periods	ΔE Mean
1d	4.21±2.9^a^
3d	4.38±2.6^a^
1w	4.42±2.8^a^
2w	4.52±3.3^ac^
1m	5.83±5.1^ade^
3w	6.15±3.0^bd^
4m	7.03±5.2^bce^
2m	7.34±5.9^be^
3m	7.69±5.4^b^

ΔE: color change. CH: calcium hydroxide, DAP: double antibiotic paste, TAP: triple antibiotic paste, TAPM: TAP Modified. d: day, m: month, w: week. Treatment (P=0.019). Period (P<0.001). Equal letters indicate statistic equivalence.

Considering only experimental periods, the ΔE means were equivalent and the lowest until the second week. On the other hand, the highest means were found at the last 3m (Table 1).

### Phase 2: Cervical sealing material

**Figure 3:**
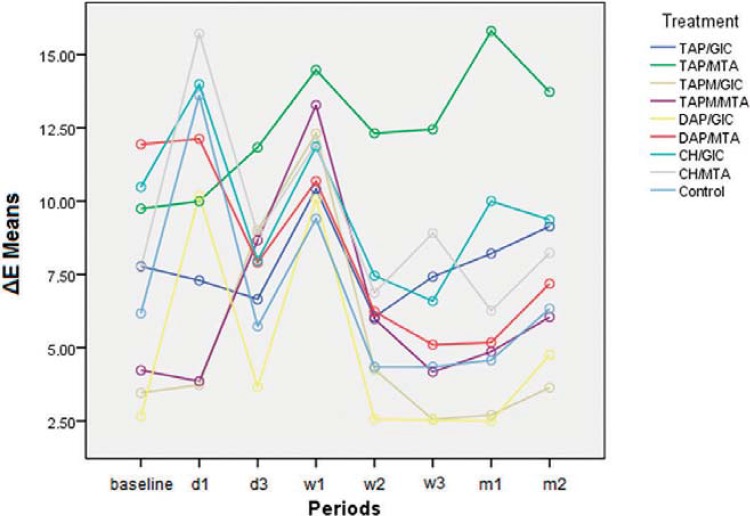
Graphic illustrating ΔE means from each group and period throughout Phase 2

There was significant difference in the interaction between treatment and periods (P<0.001). Thus, results from these variables are presented together (Table 2). At baseline, ΔE means for different groups were different due to the effect of intracanal medication in Phase 1. Only at 2w, ΔE means of TAP/GIC and TAP/MTA groups were statistically different (P=0.005). Considering absolute values, from 3d to the end of this Phase, TAP/MTA group presented the highest ΔE means, while, at the end, TAPM/GIC presented the lowest one. In the groups in which the pastes were associated with GIC, there was a tendency to lower ΔE means than in those groups in which MTA was used. At some time points, TAPM/GIC and DAP/GIC groups presented ΔE means below the human perception threshold (Table 2, Figure 3).

**Table 2:** Mean values of ΔE and standard deviation in Phase 2 (Treatment*Periods) and in Phase 3 (Treatment*Bleaching)

	TAP		TAPM		DAP		CH		Control
	GIC	MTA	GIC	MTA	GIC	MTA	GIC	MTA	
Period/th>	Phase 2. Cervical Sealing								
Baseline	7.77±0.9^abA^	9.74±5.8^abA^	3.46±1.3^aA^	4.23±2.2^acA^	2.66±1.4^abA^	11.94±11.0^abA^	10.48±5.3^acfA^	7.73±4.6^acdA^	6.17±2.7^acA^
1d	7.29±1.3^abAF^	10±5.5^aACF^	3.73±2.6^aA^	3.85±0.9^aA^	10.18±1.5^aAE^	12.12±3.8^aDEF^	13.98±1.8^aDEF^	15.71±3.3^aBCE^	13.61±3.4^bBCE^
3d	6.65±4.4^aAE^	11.83±3.2^abA^	9.01±2.0^cAC^	8.66±1.6^cAD^	3.66±1.5^bBCDE^	7.9±2.5^bcAE^	7.96±2.4^bcAE^	8.88±3.2^bcAE^	5.72±1.3^aBCDE^
1w	10.41±6.6^bA^	14.47±4.1^abA^	12.31±0.3^bA^	13.27±0.6^bA^	10.07±1.3^acA^	10.68±5.5^acdA^	11.86±1.3^adA^	11.96±2.6^acA^	9.4±2.6^cA^
2w	6.04±2.7^aA^	12.31±5.0^abBC^	4.26±0.3^aA^	5.98±0.8^aA^	2.56±0.7^bA^	6.23±1.7^bA^	7.45±2.4^beAC^	6.87±2.6^bdA^	4.34±1.5^aA^
3w	7.42±0.4^abAB^	12.45±5.2^abA^	2.55±1.1^aB^	4.17±1.0^acB^	2.53±1.3^bB^	5.1±2.9^bdAB^	6.59±3.0^bdfAB^	8.91±8.5^bcAB^	4.34±1.6^acB^
1m	8.21±2.0^abAC^	15.8±9.4^bA^	2.70±0.4^aBC^	4.86±0.7^acBC^	2.49±0.5^bBC^	5.18±2.3^bdBC^	10±3.6^acefAC^	6.26±2.9^bcBC^	4.57±1.7^aBC^
2m	9.13±1.8^abAC^	13.72±5.5^abA^	3.63±1.4^aBC^	6.04±1.8^acBC^	4.75±2.2^bcBC^	7.18±2.0^bdBC^	9.36±3.3^acefAC^	8.23±3.1^bcAC^	6.33±2.1^acBC^

**Bleaching**	**Phase 3. Bleaching**								

Baseline	9.13±1.8^aAB^	13.72±5.5^aA^	3.63±1.4^aB^	6.04±1.8^aB^	4.75±2.2^aB^	7.18±2.0^aB^	9.36±3.3^aAB^	8.23±3.1^acAB^	6.33±2.1^aB^
B1	5.05±2.4^bA^	6.87±5.2^bA^	4.76±2.5^aA^	4.19±0.7^aA^	4.88±2.2^aA^	7.1±3.8^aA^	5.54±2.1^bA^	6.74±1.5^aA^	5.93±1.3^aA^
B2	4.93±2.1^bA^	6.48±3.4^bA^	5.59±2.2^aA^	4.65±08^aA^	6.88±1.5^aA^	6.33±5.5^aA^	4.19±1.5^bA^	9.87±4.9^bcA^	6.67±2.3^aA^

ΔE: color change. B1: bleaching 1, B2: bleaching 2. CH: calcium hydroxide, DAP: double antibiotic paste, TAP: triple antibiotic paste, TAPM: TAP modified. GIC: glass ionomer cement, MTA: mineral trioxide aggregate. d: day, m: month, w: week. For comparisons, lowercase letters were used within the columns and uppercase letters within the rows. Equal letters indicate statistic equivalence. Phase 2: Treatment*Period (P<0.001). Phase 3: Treatment*Bleaching (P<0.001).

### Phase 3: Bleaching

Significant difference was found in the interaction between treatment and bleaching (P<0.001), as presented in Table 2. At baseline, ΔE means of this Phase 3 were different due to the effect of the materials used in Phase 2. There was a tendency to ΔE mean decrease after bleaching. After the first session, TAP/MTA ΔE mean decreased and showed equivalence with other groups’ outcomes. No difference was found comparing ΔE means with the first and the second bleaching sessions within the same group.

**Figure 4:**
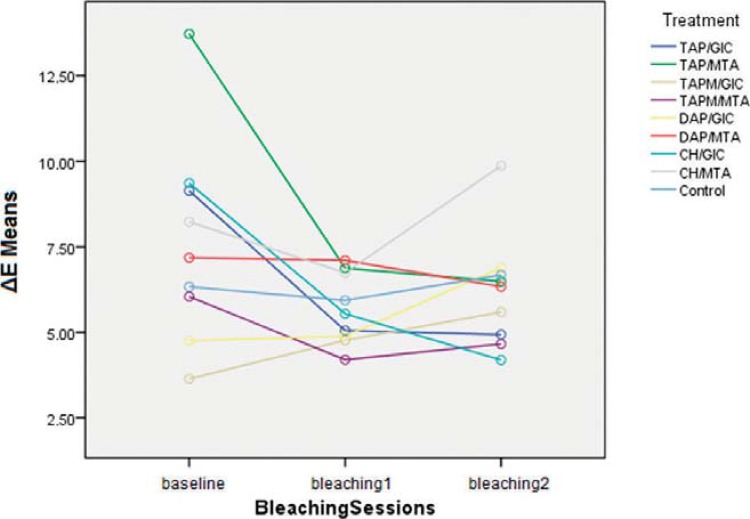
Graphic illustrating ΔE means from each group and period throughout Phase 3

It is worth pointing out that in CH/MTA group the ΔE mean after the second bleaching increased and became higher than at baseline (Figure 4, Table 2). Despite the numerical increase in ΔE mean, color shades according to VITA Classical A1–D4^®^ Shade Guide did not change in most samples (Supplemental Table 1).

## Discussion

Proposed as a therapy for immature permanent teeth, regenerative endodontic procedures have been related to tooth discoloration^10,16,23^, which could eventually lead to psychological implications^26^. So, it is important to search for treatments esthetically predictable.

Although TAP provides suitable antibacterial effect^12^, the minocycline in its composition has been associated with dental staining^9,16,23^. Thus, researchers have looked for alternatives to TAP composition^14,24,27^. In this study, TAP, TAPM (amoxicillin replacing minocycline), DAP (TAP without minocycline) and CH pastes were used. In Phase 1, TAP showed highest color alteration, while TAPM showed the lowest one. These results suggest that replacing minocycline with amoxicillin can lead to minor dental color change, improving esthetic results of REP. When minocycline was abolished (DAP), color change was lower than in TAP and higher than in TAPM groups. Although no statistically significant difference was found between DAP and TAPM, regarding tooth color stability, results suggest that antibiotic replacing is better than its suppression. However, this result was different from that found by Akcay, et al.^1^(2014), in which “TAP with amoxicillin” showed higher ΔE means than DAP group. Nevertheless, while in this study the pastes were prepared according to AAE^2^ recommendations (0.1 mg/mL), in the study of those authors the final concentration of pastes is unknown.

All groups showed variations in ΔE means through all experimental phases, and even Control group showed some color change. This result may be explained by hydration/dehydration of dental structure, which can lead to color alterations. Dehydration would result in replacement of water by air around the enamel prisms. At a dehydrated enamel-air interface, the refractive and scattering indices are different from those of enamel-water interface. Dehydrated enamel would show lower translucency causing more reflection, thus masking the underlying dentine shade, leading to a lighter appearance^8^. In this study, to reduce the effect of water loss during procedures, the time for rehydration was established in 24 h prior to color measurements. Even being careful to remove just the excessive moisture from the external dental surface and short manipulation time (±1 minute) to take color shade, some color alteration was expected^8,25^, since the major color change in dehydrated teeth occur in the first 10 minutes^8^.

When cervical sealing was performed, dental structure was under the effect of intracanal medication used in Phase 1. Thus, in Phase 2 the groups started from different values. It was observed that from 3d until the end of this Phase, the highest color alterations were registered for TAP/MTA. Although significant difference was found between TAP/MTA and TAP/GIC only at 2w, this result is especially interesting since the association TAP/MTA has been largely used in clinical practice. It is worth pointing out that in the groups in which pastes were associated with GIC, generally, ΔE means were lower than those found when they were associated with MTA, although no significant difference was found. This was expected since studies have reported that even white MTA can lead to dental staining^11,13^. The results of this study confirm the AAE^2^advice of applying GIC instead of MTA as cervical sealing material in areas where there is an esthetic concern.

In this study, a 37% carbamide peroxide gel was applied inside and outside crown structure according to manufacturer’s recommendations. This technique is largely used to bleach non-vital teeth. Although REP aims to form new vital tissue within the root canal, the coronal pulp space remains empty. This particular situation allows treating “vital teeth” by a technique indicated to non-vital teeth, because cervical sealing materials protect the vital tissue from the bleaching agent.

Interestingly, it was observed that the ΔE means of TAP/MTA group became similar to the other groups after the first bleaching session. Furthermore, except for CH/MTA group, no difference was found after one or two bleaching sessions. This suggests that, under these experimental conditions, only one session might be enough to improve tooth color. This result is clinically important considering the number of dental interventions and appointments, which reflects in treatment costs.

Despite the fact that there was a reduction in ΔE mean after the first bleaching session in CH/MTA group, it turned to increase in such a way that, after the second session, the mean ended higher than the registered at the baseline of this Phase. This may be explained by two hypotheses. First, the presence of oxygen in all materials used (CH, MTA, and carbamide peroxide) may be related to the overoxidation of bismuth oxide^15^, which is the radiopacifier of MTA. Bismuth oxide has been related to tooth staining when in contact with dentin matrix collagen^19^. Another explanation may be the interactions among the different materials used, which could produce formaldehyde as a byproduct of chemical reactions. Bismuth oxide and calcium oxide could stain dental structure when in contact with formaldehyde^4^. However, even with the increase in ΔE mean, tooth shades, according to VITA Classical A1–D4^®^ Shade Guide, did not change between the first and the second bleaching. It is possible since small color difference may not determine the change of a shade for another, as there is an interval in referential values of L*, a*, and, b* between shades^22^.

A digital spectrophotometer was used to register tooth shades along the three experimental phases. From this device, it is possible to obtain values of “L”, “a” and “b” of the dental structure and, then, calculate ΔE between time points. Spectrophotometers became largely used in Dentistry due to their reliable method of reading tooth color shade. Here, Vita Easyshade^®^ was used as it has high reliability and accuracy^17^. Additionally to the numerical information of lightness and hue, this device provides shades according to VITA Classical A1–D4^®^ Shade Guide, a standard and worldwide reference system in tooth shade determination. To further avoid external influences, silicone matrices were customized to ensure standardized area and light conditions along the experimental phases^5^. The selection of teeth with similar dentinal wall thickness is also important to improve standardized conditions^18^ and, consequently, to improve reliability of the comparisons along the time.

Here, it was investigated the effect on dental crown discoloration of products used as intracanal medication or cervical sealing in regenerative endodontics procedures, as well as the impact of dental bleaching after regenerative approach. Under limitations of an *ex vivo* study, the methodology was designed to simulate procedures performed in clinics, applying sequentially products on the same tooth to assess, throughout the experimental phases, the impact of each of them on tooth color shade.

## Conclusion

From the results of this experimental *ex vivo* study, we conclude that the evaluated intracanal medications, which are applied in regenerative endodontic approaches, influence tooth discoloration over time, being TAP the material that induces higher color alteration. On the other hand, cervical sealing material has no influence on tooth discoloration. Furthermore, dental bleaching was able to recover, at least partially, tooth color shade, mainly at first application.
